# Association Between Upper Limb Dysfunction and Six-Month Postoperative Pain Following Mini-Open Synovectomy for Lateral Epicondylitis: A Retrospective Cross-Sectional Study

**DOI:** 10.7759/cureus.101829

**Published:** 2026-01-19

**Authors:** Kazuyo Tsukamoto, Yuki Hiraga, Takamichi Taniguchi

**Affiliations:** 1 Department of Occupational Therapy, International University of Health and Welfare Graduate School, Fukuoka, JPN; 2 Department of Rehabilitation, Fukuoka Sannō Hospital, Fukuoka, JPN; 3 Department of Health Sciences, International University of Health and Welfare Graduate School, Fukuoka, JPN; 4 Department of Occupational Therapy, Fukuoka International University of Health and Welfare, Fukuoka, JPN; 5 Department of Occupational Therapy, International University of Health and Welfare Graduate School, Narita, JPN

**Keywords:** clinician-rated elbow function, lateral epicondylitis, mini-open synovectomy, patient-rated elbow evaluation, postoperative pain, upper limb function

## Abstract

Background

Refractory lateral epicondylitis (LE) often requires surgical intervention; however, the extent to which postoperative upper limb dysfunction contributes to chronic pain remains unclear. In this study, we aimed to investigate the association between postoperative upper limb dysfunction and pain intensity at six months following mini-open synovectomy for LE.

Methods

This single-center, retrospective, cross-sectional study included 28 elbows that met the predefined eligibility criteria between January 2020 and December 2023. The primary outcome was pain intensity at six months postoperatively, assessed using an 11-point numeric rating scale (NRS). Upper limb function at the same time point was evaluated using the Patient-Rated Elbow Outcome Measure Japanese Version (PREE-J), the Japanese Orthopaedic Association Elbow Study Group score, grip strength, and elbow joint range of motion (ROM). Model assumptions were evaluated using residual diagnostics (including Q-Q plots and the Shapiro-Wilk test of standardized residuals). Univariate linear regression analysis was performed to examine the associations between each functional measure and pain intensity. A pre-specified multivariate linear regression model incorporating the PREE-J score and grip strength was subsequently fitted to evaluate their independent associations with the six-month NRS score.

Results

In the forced entry multivariable linear regression model, the PREE-J score was independently associated with the six-month NRS score, whereas grip strength showed no significant association. The model explained 50% of the variance in the pain intensity, with no evidence of multicollinearity.

Conclusions

At six months following mini-open synovectomy for LE, postoperative pain was independently associated with patient-reported disability as measured by the PREE-J, whereas grip strength was not significantly associated with pain intensity. Therefore, subjective functional impairment may more accurately reflect the postoperative pain burden, compared with isolated mechanical measures. A comprehensive functional assessment incorporating the PREE-J may facilitate the early identification of patients at risk of persistent pain and support the development of individualized rehabilitation strategies.

## Introduction

Lateral epicondylitis (LE), commonly known as “tennis elbow,” is a degenerative tendinopathy characterized by pain localized to the lateral epicondyle of the humerus. It is most frequently observed in individuals aged >40 years [1]. Its incidence peaks in the fifth decade of life and remains high in the sixth decade, reflecting the age-related vulnerability of the extensor tendon origin [2]. In the general population, the prevalence of LE is estimated to range approximately 1-3%, and the condition is considerably more common than medial epicondylitis in routine clinical practice [3]. Well-established risk factors include participation in racket sports, such as tennis and badminton, and repetitive gripping activities, including golf, climbing, motocross, and mountain biking. These activities impose repetitive mechanical loading on the forearm extensor musculature and tendon complex [4]. Occupational risk factors identified using the strain index include task intensity, task duration, non-neutral wrist posture, and movement velocity; however, the relative contribution of each factor to disease onset remains uncertain [5,6]. Subsequent workplace-based ergonomic analyses have corroborated these associations, highlighting ergonomic exposure as a plausible and modifiable target for prevention [6].

Beyond localized elbow pain, LE is frequently associated with substantial upper limb dysfunction that interferes with activities of daily living and occupational performance [7]. Persistent symptoms may lead to social and work-related limitations, including reduced productivity and prolonged work absenteeism, thereby exacerbating the overall burden of the condition [8]. Functional impairment appears to be more pronounced in LE than in medial epicondylitis, suggesting distinct, condition-specific patterns of disability that warrant targeted evaluation [9]. Although conservative management is regarded as the first-line treatment, approximately 90% of patients demonstrate symptomatic improvement within one year, regardless of symptom duration [10]. However, a subset of patients fails to respond adequately to nonoperative interventions and ultimately requires surgical treatment [11]. Notably, despite surgical management, up to one-fifth of patients continue to experience chronic pain, imposing a substantial personal and societal burden [12].

The International Association for the Study of Pain defines pain as “an unpleasant sensory and emotional experience associated with, or resembling that associated with, actual or potential tissue damage” [13]. Chronic pain is generally defined as pain that persists for at least three to six months beyond the expected period of tissue healing and therefore represents a clinically meaningful endpoint when evaluating postoperative outcomes in patients with LE [14]. Although a wide range of nonoperative treatment modalities, including physical therapy, corticosteroid injections, and extracorporeal shock wave therapy, have been extensively investigated [15-17], the mechanisms underlying the progression from acute postsurgical pain to chronic pain remain incompletely understood. This knowledge gap is particularly relevant in patients undergoing mini-open synovectomy, in whom the primary structural pathology is surgically addressed; however, residual functional impairment may continue to influence postoperative pain trajectories.

Current best practices increasingly advocate for the integration of clinician-rated outcomes with patient-reported measures to comprehensively capture both observable functional performance and subjective experience in elbow disorders. The Japanese version of the Patient-Rated Elbow Evaluation (PREE-J) is a validated patient-reported instrument that quantifies pain and activity difficulty on a scale of 0-100 and has demonstrated strong reliability and validity for assessing elbow-specific disability [18]. The Japanese Orthopaedic Association-Japan Elbow Society (JOA-JES) score serves as a complementary clinician-rated outcome measure, scored on a 0-100 scale, that evaluates pain, range of motion (ROM), muscle strength, and activities of daily living (ADLs), thereby enabling standardized comparisons of postoperative elbow function across clinical settings [19]. Previous studies have suggested that preoperative upper limb dysfunction may influence postoperative pain intensity and subsequent functional recovery, underscoring the contribution of functional impairment to the progression of pain following surgical intervention [20].

Against this background, in the present study, we aimed to determine whether postoperative upper limb dysfunction is associated with pain persistence six months following mini-open synovectomy for LE. Specifically, the associations were examined between pain intensity at six months, measured using the numeric rating scale (NRS), and contemporaneous functional status, assessed using the PREE-J and JOA-JES score, grip strength, and elbow ROM. By identifying functional factors independently associated with persistent postoperative pain, we aimed to facilitate early risk stratification and inform the development of individualized rehabilitation strategies to optimize postoperative recovery.

## Materials and methods

Study design and setting

This study was designed and reported in accordance with the Strengthening the Reporting of Observational Studies in Epidemiology (STROBE) guidelines. A single-center, retrospective, cross-sectional study was conducted at Fukuoka Sannō Hospital, Fukuoka, Japan, where all surgical procedures were performed. Outcome assessments were conducted in collaboration with Fukuoka International University of Health and Welfare. The study period spanned from January 2020 to December 2023.

Participants

Eligible participants were adults (aged ≥18 years) diagnosed with LE based on marked tenderness over the forearm extensor muscles and positive provocation tests, such as the Thomsen test, corroborated by magnetic resonance imaging (MRI) findings demonstrating high T2 signal intensity at the extensor origin. Patients who did not achieve improvement after at least six months of conservative therapy and completed a six-month postoperative follow-up after mini-open synovectomy performed by a single surgeon were included in the study. Patients with severe elbow cartilage lesions, rheumatoid arthritis, psychiatric or neurological disorders, inability to follow instructions, nonadherence to the postoperative protocol, and incomplete clinical records were excluded.

**Figure 1:**
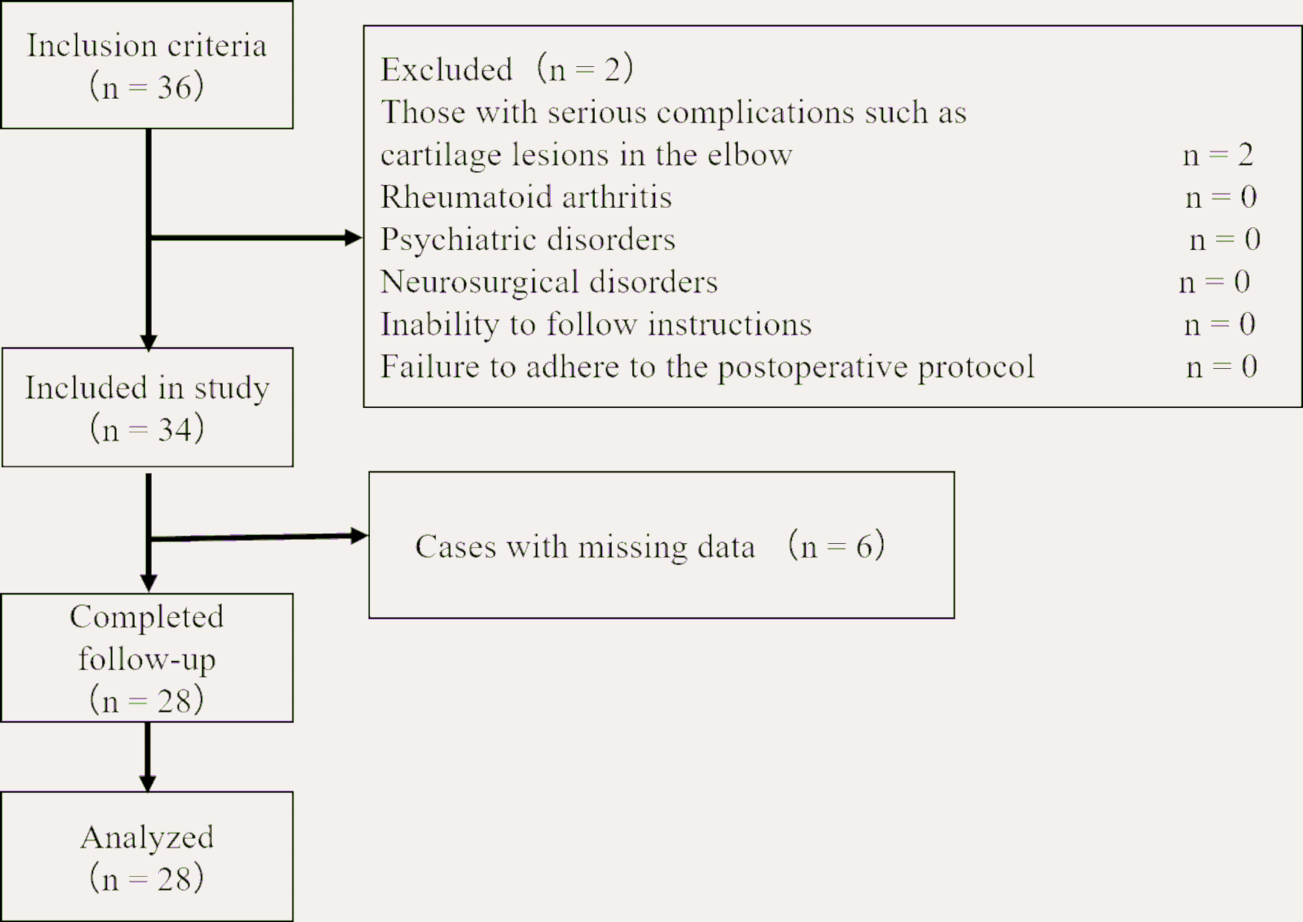
Overview of the study protocol

Of the 36 consecutive elbows screened, eight were excluded (two because of severe cartilage lesions and six because of missing data), resulting in a final sample of 28 elbows for analysis (Figure 1).

Demographic and clinical characteristics

Demographic and clinical data were extracted from medical records by attending physicians and occupational therapists at Fukuoka Sannō Hospital. Demographic variables included age, sex, body mass index (BMI), hand dominance, and affected side. Clinical variables comprised disease duration (from symptom onset to surgery), number of previous steroid injections (triamcinolone administered at doses of 10-40 mg in some patients), occupational classification, daily activity level, and imaging or surgical findings (including preoperative MRI results and synovial pathology).

Surgical approach

Patients were placed in the supine position. A 2-3-cm skin incision was made over the superior margin of the radial head. The origin of the extensor carpi radialis brevis (ECRB) was identified and excised along with the adjacent joint capsule. Additionally, approximately one-quarter to one-third of the proximal portion of the annular ligament was resected. Consequently, the lateral joint capsule, including the ECRB-extensor digitorum communis origin, was confirmed to become lax during forearm pronation and supination.

Rehabilitation protocol

All patients adhered to a standardized institutional postoperative protocol. Wound dressings were maintained until postoperative day 3, after which drains were removed, and the elbow was immobilized with a splint extending from the upper arm to the forearm for two weeks. Postoperative pain was primarily managed with a short course of nonsteroidal anti-inflammatory drugs, with acetaminophen or adjuvant analgesics administered as needed. Occupational therapy, including shoulder and finger ROM exercises and stretching, was initiated on postoperative day 3. Patients were typically discharged at approximately postoperative week 3, depending on physician approval and patient readiness. Following splint removal, elbow ROM exercises and stretching were progressively advanced, with muscle strengthening introduced as tolerated based on individual symptoms. Training in ADLs and ADL-related tasks was tailored to each patient’s functional needs.

Study outcomes

The primary outcome was pain intensity at six months postoperatively, measured using the 11-point NRS, where 0 indicates no pain, and 10 represents the worst imaginable pain [21].

Upper Limb Function at Six Months (Independent Variables)

Unless otherwise specified, all functional assessments were performed at the six-month postoperative visit. The following instruments and measurements were used.

PREE-J: A patient-reported, elbow-specific measure comprising five items assessing pain and 11 items assessing difficulty with ADLs. Total scores range from 0 to 100, with higher scores indicating greater impairment [18].

JOA-JES score: A clinician-rated composite score evaluating pain, ROM, muscle strength, and ADLs. Total scores range from 0 to 100, with higher scores indicating better function [19].

Grip strength: Maximal isometric grip strength was measured using a digital dynamometer (Takei Kiki Kogyo Grip-D, Smedley type; Takei Kiki Kogyo Co., Kamo City, Niigata Prefecture, Japan) in the seated position, with the elbow flexed at 90° and the forearm in a neutral position. To minimize participant burden, a single maximal trial was performed for each upper limb.

Elbow ROM: Flexion and extension were measured using a goniometer in the seated position, with the upper arm stabilized against the trunk and the radial shaft serving as the reference axis.

All six-month functional assessments were performed by experienced staff who were blinded to the study hypotheses.

Ethical considerations

This retrospective study was approved by the Ethics Review Committee of Fukuoka Sannō Hospital (approval no. 24-KS-015). De-identified data were extracted from medical records, with no direct patient contact or intervention involved. In accordance with institutional policy and applicable regulations, the requirement for individual informed consent was waived by the committee. All data were managed in compliance with institutional data protection policies and the principles outlined in the Declaration of Helsinki. No identifiable images or personal information were included in this article.

Statistical analysis

All analyses were conducted using complete case data. Continuous variables were initially assessed for normality using the Shapiro-Wilk test. Univariable linear regression analyses were performed with the six-month NRS score as the dependent variable and each functional measure as an independent variable. For multivariate analysis, a forced entry multivariable linear regression model was fitted with the six-month NRS score as the dependent variable. The candidate independent variables were pre-specified as the PREE-J score and grip strength to examine the independent contribution of patient-reported disability while accounting for physical impairment. This pre-specified approach was selected to minimize the risk of overfitting associated with automated variable selection methods. Multicollinearity was evaluated using the variance inflation factor (VIF), with a value <10 considered acceptable. The normality of standardized residuals was evaluated using Q-Q plots and the Shapiro-Wilk test. Statistical significance was defined as a p-value of <0.05. All analyses were conducted using IBM SPSS Statistics for Windows, version 31 (Released 2025; IBM Corp., Armonk, New York, United States).

## Results

Participant characteristics

The baseline characteristics of the cohort are summarized in Table 1. The cohort comprised 14 male and 14 female participants (male-to-female ratio, 1:1), with a mean age of 51.1 ± 8.0 years. Of the participants, 27 were right-handed, whereas only one was left-handed. The procedure was performed on the right side in 17 participants and on the left side in 11. The mean disease duration was 26.9 ± 23.6 months, whereas the mean number of previous steroid injections was 5.1 ± 4.0, indicating that surgery was generally preceded by an extended course of conservative management.

**Table 1:** Baseline characteristics of the study Values are expressed as the mean ± SD unless otherwise indicated. BMI: body mass index; PREE-J: Patient-Rated Elbow Evaluation-Japanese version; JOA-JES: Japanese Orthopaedic Association-Japan Elbow Society; ROM: range of motion; NRS: numeric rating scale

Variable		Value
Age (years)		51.1 ± 8.0
Female sex, n (%)		14 (50)
Surgical side, right, n		17
Dominant hand right, n (%)		27 (96)
Disease duration (months)		26.9 ± 23.6
Number of prior steroid injections		5.1 ± 4.0
BMI (kg/m^2^)		24.0 ± 3.4
PREE-J score		56.4 ± 20.3
JOA-JES score		33.1 ± 14.9
Grip strength (kg)		18.5 ± 11.5
Active flexion ROM (°)		137.1 ± 10.1
Passive flexion ROM (°)		141.4 ± 7.6
Active extension ROM (°)		−2.5 ± 10.0
Passive extension ROM (°)		1.6 ± 7.7
NRS score		8.0 ± 2.0

Association between upper limb function and pain (univariable analyses)

Univariable linear regression analyses were conducted with the six-month NRS score as the dependent variable and each functional measure as an independent variable (Table 2). The JOA-JES score demonstrated a significant negative association with the NRS score (standardized β = −0.717, 95% confidence interval (CI) for slope = −0.091 to −0.039; p = 0.001; R² = 0.514). Grip strength also demonstrated a significant negative association (standardized β = −0.490, 95% CI for slope = −0.177 to −0.029; p = 0.008; R2 = 0.241). Similarly, the PREE-J score showed a significant positive association with the NRS score (standardized β = 0.706, 95% CI for slope = 0.059 to 0.139; p < 0.001; R² = 0.498). In contrast, elbow ROM was not significantly associated with the NRS score in the univariate analysis. These findings suggest that higher clinician-rated functional recovery (JOA-JES) scores and greater grip strength were linked to lower postoperative pain, whereas higher patient-reported disability (PREE-J) scores corresponded to higher postoperative pain. Simple ROM alone did not account for variability in pain in these analyses.

**Table 2:** Results of the simple regression analysis β: standardized coefficient; 95% CI: 95% confidence interval; PREE-J: Patient-Rated Elbow Evaluation-Japanese version; JOA-JES: Japanese Orthopaedic Association-Japan Elbow Society; ROM: range of motion; NRS: numeric rating scale

Dependent variable	Independent variable	β	95% CI	p value
6-month postoperative NRS score	Constant	−0.717	−0.091 to −0.039	<0.001
	6-month postoperative JOA-JES score			
	R^2 ^= 0.514			
6-month postoperative NRS score	Constant	0.706	0.059 to 0.139	<0.001
	6-month postoperative PREE-J score			
	R^2 ^= 0.498			
6-month postoperative NRS score	Constant	−0.490	−0.177 to −0.029	p = 0.008
	6-month postoperative grip strength (kg)			
	R^2 ^= 0.241			
6-month postoperative NRS score	Constant	−0.023	−0.168 to 0.15	p = 0.909
	6-month postoperative active flexion ROM (°)			
	R^2 ^= 0.0005			
6-month postoperative NRS score	Constant	0.267	−0.177 to 0.033	p = 0.170
	6-month postoperative active extension ROM (°)			
	R^2 ^= 0.071			

Association between upper limb function and pain (multivariable analysis)

A multiple linear regression analysis using the forced entry method was conducted with the six-month NRS score as the dependent variable and the PREE-J score and grip strength as independent variables (Table 3). The PREE-J score was significantly associated with the 6-month NRS score (B = 0.085, 95% CI = 0.042-0.128; β = 0.608; p < 0.001), whereas grip strength was not (B = −0.046, 95% CI = −0.112 to 0.020; β = −0.218; p = 0.164). The model’s adjusted R² was 0.500, indicating that it accounted for 50% of the variance in postoperative pain. Multicollinearity was negligible (VIF = 1.25 for both variables).

**Table 3:** Results of the multivariable linear regression analysis B: unstandardized regression coefficient; β: standardized coefficient; 95% CI: 95% confidence interval; VIF: variance inflation factor; PREE-J: Patient-Rated Elbow Evaluation-Japanese version; NRS: numeric rating scale

Dependent variable	Independent variable	B	95% CI	β	p value	VIF
6-month postoperative NRS score	6-month postoperative PREE-J score	0.085	0.042 to 0.128	0.608	<0.001	1.25
	6-month postoperative grip strength (kg)	−0.046	−0.112 to 0.020	−0.218	0.164	1.25

## Discussion

Although chronic pain is typically defined by duration, the present study evaluated pain intensity at a single postoperative time point (six months), addressing pain persistence rather than the processes underlying pain chronification. This study investigated the association between upper limb function and pain intensity six months after mini-open synovectomy for LE. To account for the limited sample size and minimize the risk of overfitting, a pre-specified multivariable model was employed, focusing on two key functional domains: patient-reported disability (PREE-J) and physical impairment (grip strength). In this model, the PREE-J score was identified as a significant independent predictor of the six-month NRS score, whereas grip strength was not a significant predictor. The model demonstrated a moderate explanatory power (adjusted R^2^=0.500) and no evidence of multicollinearity (VIF = 1.25); however, these results should be interpreted with caution, as the deliberate restriction of candidate variables was necessary due to the small cohort. Nevertheless, these findings suggest that persistent postoperative pain may be more closely related to patient-reported disability than to isolated physical parameters such as grip strength.

Most previous studies on LE have focused on the efficacy of conservative treatments and the analgesic effects of surgical intervention, with psychological and histopathological factors frequently proposed as contributors to pain chronicity [11,12]. However, quantitative analyses of postoperative functional outcomes remain limited. The present findings complement the reports of previous studies by suggesting that preoperative dysfunction may influence the persistence of postoperative pain [15] and further demonstrating that postoperative functional status at six months, specifically patient-reported disability assessed using the PREE-J, is significantly associated with pain intensity at the same time point. These results are directionally consistent with observations in nonoperative settings, where pain and functional impairment are closely interrelated in chronic LE [22]; they extend this relationship to a surgical population at a clinically relevant mid-term follow-up.

From a clinical perspective, incorporating both the JOA-JES score and PREE-J into routine postoperative follow-up may facilitate early identification of patients at risk for persistent pain and guide the development of individualized rehabilitation strategies. As patient-reported disability provides insights beyond that captured by clinician-rated assessments, postoperative care should integrate impairment-focused interventions, such as ROM restoration, progressive strengthening, and task-specific motor retraining [23], with strategies addressing activity limitations and participation, including education, activity pacing, and graded functional exposure [24,25].

However, this study has some inherent limitations that warrant careful consideration when interpreting these findings. First, this was a single-center study with a modest sample size (n = 28), limiting the statistical power for complex multivariate modeling and restricting the generalizability of our results. Psychosocial factors (e.g., anxiety, depression, and fear-avoidance beliefs) were not assessed, preventing a comprehensive evaluation of the biopsychosocial mechanisms that may mediate or moderate postoperative pain trajectories. Outcomes were measured only at six months, leaving longer-term trajectories (e.g., one to two years) undefined. Furthermore, all surgical procedures were performed by a single surgeon within a standardized institutional rehabilitation framework, ensuring treatment consistency but potentially limiting generalizability.

Prospective, multicenter studies with larger cohorts, longer follow-up periods, and integrated assessments of psychosocial factors are warranted to validate these findings and determine whether early, multidimensional functional assessments can enhance prognostic accuracy and guide targeted, individualized interventions.

In summary, the findings indicate that postoperative pain six months following mini-open synovectomy for LE is more closely associated with patient-reported functional status (assessed using the PREE-J) than with isolated physical measures, such as grip strength. These findings underscore the value of a postoperative evaluation approach that incorporates multidimensional functional assessment to identify patients at risk for persistent pain and guide the development of individualized rehabilitation strategies.

## Conclusions

Six months after mini-open synovectomy for LE, postoperative pain intensity was independently associated with patient-reported disability (assessed using the PREE-J), whereas grip strength was not a significant predictor in the forced entry multivariable model. These findings indicate that persistent postoperative pain is more linked to subjective functional status, reflecting the patient’s perception of disability, than to isolated mechanical measures such as grip strength.

From a clinical perspective, the routine incorporation of comprehensive assessments, particularly the PREE-J, may facilitate early risk stratification and guide individualized rehabilitation that integrates impairment-based interventions with patient-centered strategies, including education, activity pacing, and graded functional exposure. Prospective, multicenter studies with longitudinal follow-up and the inclusion of psychosocial variables are warranted to further validate these associations and determine whether early, comprehensive assessment can reduce chronic postoperative pain.
